# Dual Cluster Head Optimization of Wireless Sensor Networks Based on Multi-Objective Particle Swarm Optimization

**DOI:** 10.3390/s23010231

**Published:** 2022-12-26

**Authors:** Aiyun Zheng, Zhen Zhang, Weimin Liu, Jiaxin Liu, Yao Xiao, Chen Li

**Affiliations:** College of Mechanical Engineering, North China University of Science and Technology, Tangshan 063210, China

**Keywords:** wireless sensor network, cluster head, energy consumption, multi-objective optimization, mobile sink

## Abstract

Energy conservation is one of the main problems in a wireless sensor network (WSN). Compared with a single cluster head (CH), a dual CH optimization was proposed for less energy consumption by the WSN and an acquisition delay by the mobile sink (MS). Firstly, a fuzzy c-means clustering algorithm and a multi-objective particle swarm optimization were utilized for the determinations of the first and second CHs. Following that, the ideal trajectory of MS was assessed using the improved ant colony algorithm. Finally, the lifetimes, the death rounds of the first node and the 50% node, and the number of packets received at the base station were compared among the proposed approach. Moreover, five algorithms were compared to validate the optimization, and the improved trajectory was compared with the original one as well. It was found that, for 100 nodes, the number of dead rounds from the proposal increased by 7.9%, 22.9%, 25.1%, 61%, and 74.4% for the first node, and that of the 50% nodes increased by 27.8%, 34.2%, 98.3%, 213.1%, and 211.2%, respectively. The base station packet reception increased by about 19.3%, 53.5%, 27%, 86.8%, and 181.2%, respectively. The trajectory of MS could also decrease by about 10%.

## 1. Introduction

The internet of things (IOT) is widely applied in people’s daily lives, such as through smart homes, smart health care, smart agriculture, and rescue monitoring in Refs. [1,2,3,4]. As an essential part of IOT applications, a wireless sensor network (WSN) functions via massive deployed sensors in wireless communication. Usually, node energy consumption decides the WSN’s life from the perspectives of native restraints and battery energy maintenance (Ref. [5]).

Representing an important technology for prolonging the network lifetime, clustering is known in most research. Ref. [6] suggested that an appropriate cluster head (CH) could reduce the sensor’s energy consumption. In 2019, Ref. [7] adopted K-means and a fuzzy analytic hierarchy process for CH based on transmission distance, energy, and hop number and reported the effectively extended network life. Ref. [8] applied fuzzy inference to the CH selection, and designed two different clustering approaches with a threshold for the improved network lifetime. To reduce the load on CH, Ref. [9] applied the assistant CH and the super CH to aggregate and transmit data for the improved lifetime of CH, respectively.

As for the energy consumption of nodes, data transmission was considered the main influencing factor by Ref. [10]. Regarding the process of data transmission, Ref. [11] proposed that relay nodes could save energy by shortening distances between the nodes and the sink node. Moreover, the data could be aggregated by the relay node to reduce the amount of transmitted data, eliminate data redundancy, reduce energy consumption, and prolong the network life (Ref. [12]). Typically, nodes send data to the base station (BS) in multi-hop, which may bring hotspot problems for the nodes near the BS in WSNs. To avoid the hotspots in data receiving, a mobile sink (MS) is an effective and popular method. However, Refs. [13,14] claimed that, in data collection, the inappropriate trajectory of MS may lead to a rise in data collection delay. Facing the same problem, Ref. [15] believed that multiple data rendezvous points could collect data from other nodes while MS collected data from rendezvous points. According to the reference, the rendezvous points transmitting data from clusters acted as CHs, and a more ideal trajectory of MS between CHs could effectively reduce the collection delay on some level.

### 1.1. Objects

The objects of this paper are listed as follows:

- Fuzzy c-means (FCM) algorithm with the optimal cluster number is utilized to obtain the uniformly distributed first cluster heads (FCHs) for the energy conservation of nodes;
- Based on the FCHs, a multi-objective particle swarm optimization (MOPSO) is applied for the second cluster heads (SCHs) to lessen the load on the FCHs, and its search speed is controlled by the normal distribution decay inertial weight and the sigmoid-based acceleration coefficients to avoid a locally optimal solution;
- With the selected SCHs, the improved ant colony optimization (ACO) is capable of seeking the shortest trajectory of MS for reduced delay;
- The effectiveness of the proposed approach is examined by the comparisons of the lifetime, the death times of the first node and 50% nodes, and the trajectory length of MS.

### 1.2. Related Work

Commonly, the greater distance between the nodes and sink burdens the energy consumption of data transmission in WSNs. To solve this problem, network clustering was preferred by Refs. [16,17,18]. As a benchmark for the performance of WSNs, the essentials of low-energy adaptive clustering hierarchy (LEACH) were to divide the entire network into several clusters for random CHs. Since the residual energy of the node is not considered by LEACH, its application would lead to an uneven distribution of CHs. According to Ref. [19], the energy consumption of some nodes intrigued such excessive premature death that the selection of CH was quite important.

According to Refs. [20,21,22,23,24], the data collection of MS can solve the energy hole in WSNs with two implementations. One is the direct collection: the MS directly collects data from all sensor nodes to solve the hotspot problem, but sometimes the data collection is severely delayed due to the longer trajectory length brought on by too many nodes. The other one is to collect data from common nodes via CH in the cluster. Although MS only collects data from a few CHs instead of all sensors, it may still engage with some small-scale hotspots, CH in detail, through frequent access.

When it came to the second implementation, metaheuristic approaches were reported as effective in the CH selection and the trajectory formation.

To obtain a suitable CH, Ref. [25] proposed the improved ACO of MS based on the CH distance factor and discovered that the first dead nodes were unveiled around 2600 rounds as all nodes died at around 3400 rounds. In 2018, Ref. [26] proposed an MS energy-saving routing approach grounded on particle swarm optimization (PSO) by the node weight and applied a PSO for a set of the optimal CHs. They found that for 20 and 30 nodes under a tour length of 100 m, the energy consumption of nodes after 49 rounds was about 0.00055 J and 0.00083 J, respectively. Moreover, an energy-saving CH selection via MS was proposed by Ref. [27]: compared with traditional technologies, the proposed approach improved the network lifetime by about 40%. In addition to those, Ref. [28] applied the BAT algorithm for WSNs. In their work, the fitness function was constructed using the ratio of residual energy to the distance between node and CH for the optimal CH combination. When the initial energy was 0.1 J, the dead nodes of the network began to appear in 78 rounds and all died in 207 rounds. 

Usually, after the CH selection, the formation of the optimal trajectory is the next issue to be determined. Ref. [29] employed a PSO for the trajectory of MS: the process of MS visiting CH was regarded as a traveling salesman problem (TSP). In addition, the reverse order strategy and simulated annealing were applied to improve the PSO. In the case of 100 nodes with a mutual communication range of 50 m, the trajectory was reduced by about 25% after optimization. In 2019, Ref. [30] proposed a data acquisition using MS. In their work, the unmanned aerial vehicle was treated as an MS, and after clustering via the dynamic clustering algorithm and the CH with the highest energy, the combination of the ACO and angular bisector algorithms optimized the trajectory by 13.4%. Ref. [31] applied a genetic algorithm and PSO for the CH selection and the optimal route, respectively, and reported that, for 100 and 200 nodes, the network began to die at around 1300 and 1100 rounds, respectively, and all nodes died at around 3500 rounds.

The rest of this paper is as follows: Section 2 introduces the system model and the energy model. Section 3 introduces the dual CH approach and the details of the CH selection. Section 4 gives the simulation comparison results. Section 5 concludes.

## 2. Problem Formulation

### 2.1. System Model

The following prerequisites were assumed for this approach:

- The network has n sensor nodes deployed in an *F* × *F* area.
- All nodes acknowledge the location of BS.
- The sensor nodes are fixed once they are deployed.
- Each sensor node possesses a unique ID and position awareness.
- The communication range is set as the same between all sensor nodes and MS.
- Nodes should send data to the CH of a cluster in a single hop.
- The speed of MS is fixed as *v* with infinite energy.
- All sensor nodes are isomorphic, and energy is limited.

**Figure 1:**
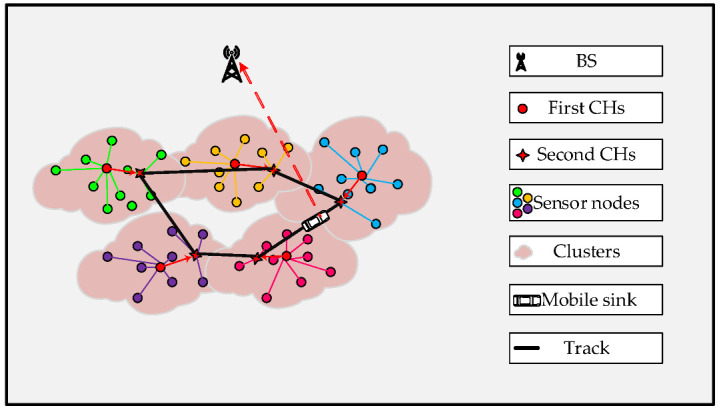
Data collection.

The detailed description of the data collection process is plotted in Figure 1 and was introduced as follows: Firstly, the FCH was selected before setting a group of SCHs. After that, according to the distance size, the cluster member nodes were chosen to transmit data to the FCHs or SCHs. The data aggregated through the FCHs was transmitted to the SCHs. Finally, MS visited SCHs via the optimal trajectory to send the data to BS.

### 2.2. Energy Model

**Figure 2:**
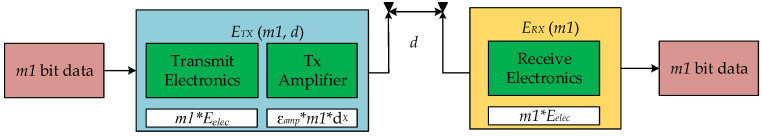
Radio energy consumption model.

The energy consumption of the node sending or receiving data determines the form of the energy consumption equation under data transmission distance *d*. The whole energy model is shown in Figure 2. When *d* is less than or equal to *d*_0_, the energy consumption is predicted by Formula (1). When *d* is greater than *d*_0_, the energy consumption relies on Formula (2). The *d*_0_ mentioned above is written as Formula (3). In addition to those, the received energy of the node is shown in Formula (4).

(1) $$E_{TX}=m1*E_{elec}+m1*\varepsilon_{fs}*d^{2}\;\;\;d\leq d_{0}$$

In Formula (1), *E_TX_*(*m*1, *d*) represents the energy consumption of the node sending data, *m*1 is the *m*1 bit of sent data, Eelec represents electronic energy, and *ε_fs_* is the parameter in the power amplifier for the free space channel propagation model.

(2) $$E_{TX}=m1*E_{elec}+m1*\varepsilon_{mp}*d^{4}\;\;\;d>d_{0}$$

In Formula (2), *ε_mp_* is a parameter in the power amplifier for the multipath fading channel propagation model.

(3) $$d_{0}=\sqrt{\frac{\varepsilon_{fs}}{\varepsilon_{mp}}}$$

(4) $$E_{RX}=m1*E_{elec}$$

In Formula (4), the energy consumption of the node receiving data is only related to the data amount.

## 3. Proposed Energy-Efficient Dual CH Approach

The procedure of this approach was divided into four steps: 

Step 1: FCM algorithm is applied to the cluster network. 

Step 2: Energy and distance factors of the node are used to select the FCH based on the clustering results. 

Step 3: The MOPSO is utilized to select the SCH according to the outcomes of Steps 1 and 2. 

Step 4: The improved ACO is applied for the optimal trajectory of MS.

**Figure 3:**
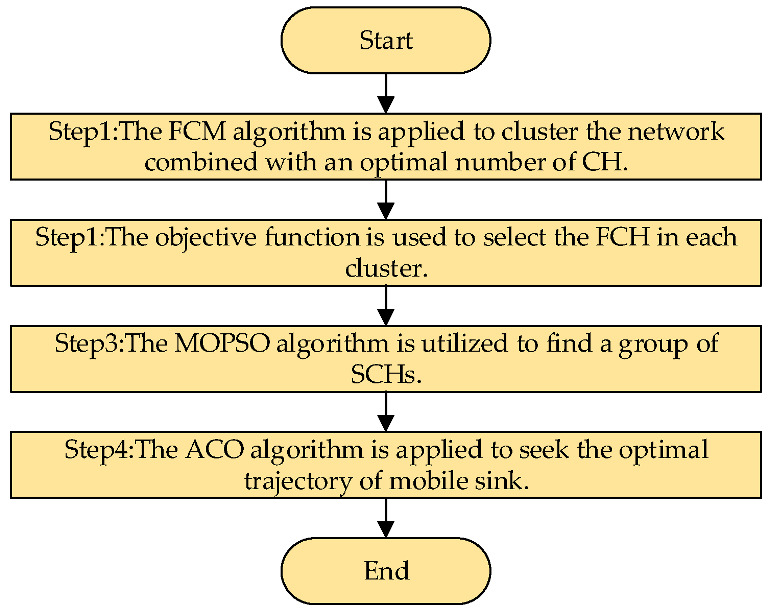
The schematic diagram of the proposed approach.

The process of the proposed approach is shown in Figure 3.

### 3.1. Network Clustering

#### 3.1.1. Number of Clusters

Considering that the number of clusters could strongly decide the quality of network clustering, three widely applied clustering approaches were compared by Ref. [32], and it was found that the rule of thumb shown in Formula (5) was most effective in energy conservation.

(5) $$k=\sqrt{\frac{n}{2}}$$

where *n* is the number of sensors.

The optimal CH number from Ref. [33] is addressed as in Formula (6).

(6) $$k=\sqrt{\frac{n}{2\pi }}*\sqrt{\frac{\varepsilon_{fs}}{\varepsilon_{mp}}}*\frac{F}{d_{toBS}^{2}}$$

In Formula (6), *F* is the edge length of the sensor square area and *d_toBS_* is the average distance from all nodes to BS.

Both Formulas (5) and (6) were employed for the CH number. The obtained *k* from Formula (5) was 7.0711, while the average one from Formula (6) was 7.5879. The other detailed outcomes of *k* in Formula (6) are listed in Table 1. Considering the fact that those two results varied between 7 and 8, the *k* in this work is set at 8.

#### 3.1.2. Fuzzy C-Means Clustering

In the process of clustering, some nodes were compulsorily assigned to specific clusters, which might cripple the energy balance in the clusters.

The network was divided into *k* categories with the FCM clustering, the fuzzy degree of each node to the cluster center $v_{j}^{o}$ was named as the membership degree. The membership degree of node *SN_i_* relative to each cluster center is expressed as *u_ij_*.

Formula (7) indicates that the sum of the membership degree of the same node *SN_i_* to the different cluster center $v_{j}^{o}$ is 1.

(7) $$\sum_{j=1}^{k}u_{ij}=1$$

The objective function is shown in Formula (8).

Where *k* is the number of clusters, *ds_ij_* represents the Euclidean distance from the node *sn_i_* to $v_{j}^{o}$, *m* is the fuzzy weighted index, and *m* = 2.

(8) $$J_{FCM}=(U,V^{O})=\sum_{j=1}^{k}\sum_{i=1}^{n}u_{ij}^{m}ds_{ij}^{2}=1$$

The membership degree is presented as Formula (9).

(9) $$u_{ij}=\frac{1}{\sum_{t=1}^{k}{\left(\frac{ds_{ij}}{ds_{ti}}\right)}^{\frac{2}{m-1}}}$$

The clustering center is listed as Formula (10).

(10) $$v_{j}^{o}=\frac{\sum_{i=1}^{n}{\left(u_{ij}\right)}^{m}sn_{i}}{\sum_{i=1}^{n}{\left(u_{ij}\right)}^{m}}$$

**Figure 4:**
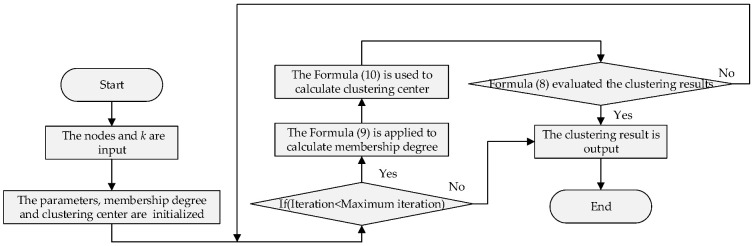
Network clustering.

The flow chart of the network clustering process is shown in Figure 4. In this procedure, the cluster number in the FCM clustering is determined by *k*. After the parameters, membership, and clustering centers were initialized, Formula (8) evaluated and finalized the clustering results. The involved membership degree and clustering center were estimated by Formulas (9) and (10).

### 3.2. Selection of FCH

During the selection of the FCH, we needed to consider the average node distance in the cluster and the node residual energy to determine the appropriate CH.

The FCH at an appropriate location is conducive to narrowing intra-cluster gaps.

(11) $$\;f_{fd}=\frac{1}{h_{sn}-1}\sum_{x=1}^{h_{sn}}Dis(SN_{X},FN)$$

where *h_sn_* represents the number of nodes in a cluster, *Dis*(*SN_X_*, *FN*) represents the distance from the non-CH node in the cluster to the FCH, and *FN* represents a certain CH.

Formula (12) was treated as the energy criterion for nodes.

(12) $$f_{fe}=\frac{\sum_{x=1}^{h_{sn}}E(x)}{E(ch)}\;ch=1,2,3\ldots k$$

where *E*(*x*) represents the residual energy of the non-CH nodes in the cluster, and *E*(*ch*) represents the residual energy of the selected CH.

**Figure 5:**
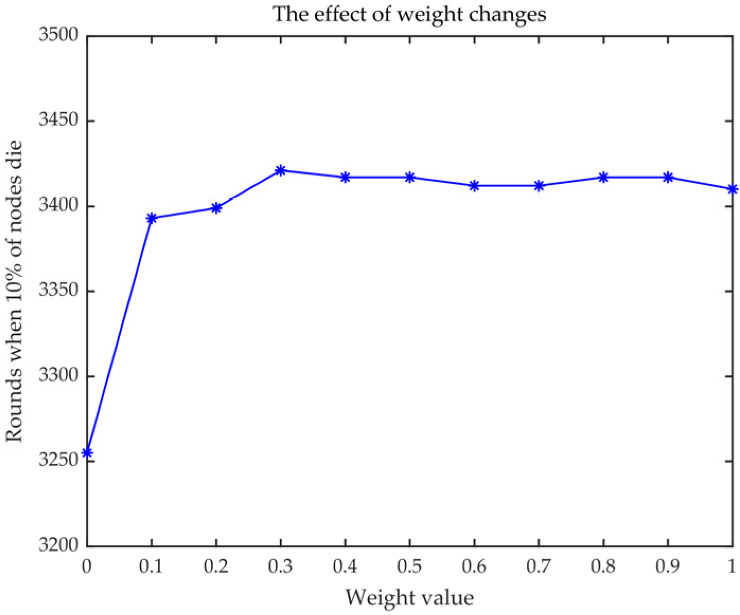
Selection weight of Formula (11).

Formula (13) was utilized to select FCH in each cluster.

(13) $$Minimize\;f_{fz}=\psi *f_{fd}+(1-\psi )*f_{fe}$$

where *Ψ* in Figure 5 is expressed as the weight of Formula (11). *Ψ* = 0.3 is confirmed for further application in this work with the maximum round of 3421.

CH shift: the objective value of each node was estimated by Formula (13). The node with the smallest objective datum in each cluster is defined as the current FCH.

### 3.3. Selection of SCH

MOPSO was used to seek the SCH according to the objective Formulas (14)–(17).

(14) $$Minimize\;f_{cd}=\sum_{s=1}^{k}\left(\frac{1}{h_{sn}}\sum_{x=1}^{h_{sn}}Dis(SN_{X},CN_{S})\right)$$

where *Dis*(*SN_X_*, *CN_S_*) represents the distance from the single non-CH node to the SCH.

(15) $$Minimize\;f_{ce}=\sum_{ch=1}^{k}\frac{\sum_{x=1}^{h_{sn}}E(x)}{E(ch)}$$

In Formula (15), *E*(*x*) represents the residual energy of the non-CH nodes in the cluster, and *E*(*ch*) represents the residual energy of SCH.

The residual energy of nodes is shown in Equation (16).

(16) E(x)=E(init)−E(cons)

where *E*(*init*) represents the initial energy of the node, and *E*(*cons*) represents the energy consumed by the node, and *E*(*cons*) is calculated by Formulas (1)–(4)

**Figure 6:**
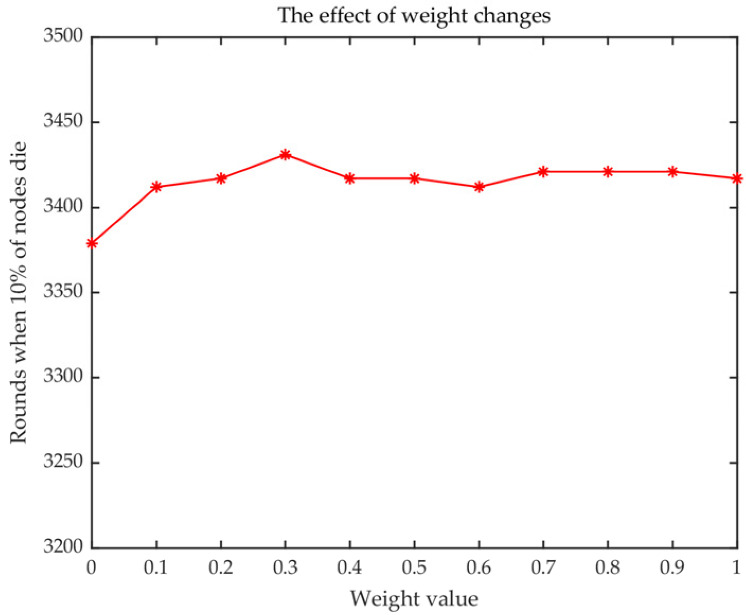
Selection weight of Formula (17).

The relationship between CHs, Formula (17), is taken as an optimization objective.

(17) $$Minimize\;f_{cf}=\frac{\lambda }{k}*\sum_{s=1}^{k}\left(Dis(FN_{S},CN_{S})\right)+\frac{\left(1-\lambda \right)}{k}*\sum_{w=1}^{k}\sum_{s=1}^{k}Dis(CN_{W},CN_{S})$$

where *Dis*(*FN_S_*, *CN_S_*) represents the distance between the FCH and the corresponding SCH, and *Dis*(*CN_W_*, *CN_S_*) represents the distance between one SCH and the others. *λ* addresses the weight of 0.3 based on Figure 6.

#### 3.3.1. Encoding

In this paper, each particle represents a group of SCHs, so the dimension of the particle is twice the number of the SCHs. Two attributes of particles were namely position *X* and velocity *V*. The position and velocity of particles were updated with each iteration.

The updated position is shown in Formula (18):

(18) $$X_{a}^{r}=X_{a}^{r-1}+V_{a}^{r-1}$$

where *X_a_^r^* represents the position of the *r*th generation of Particle *a* and *V_a_^r^*^−1^ represents the velocity of the (*r* − 1)th generation of Particle a.

The updated velocity is written as Formula (19).

(19) $$V_{a}^{r}=\omega *V_{a}^{r-1}+c_{1}*r_{1}*(pbest_{a}^{r-1}-X_{a}^{r-1})+c_{2}*r_{2}*(gbest_{a}{}^{r-1}-X_{a}^{r-1})$$

where *ω* is the inertia weight, *c*_1_ and *c*_2_ represent the acceleration coefficient, *r*_1_ and *r*_2_ represent the random numbers between [0, 1], *pbest* represents the individual leader, and *gbest* represents the global leader.

Particle velocity restriction: at the end of the iteration, *V* is compared with *V_max_* and −*V_max_* and if *V* is greater than *V_max_*, then *V* = *V_max_*, and if *V* is less than −*V_max_*, then *V* = −*V_max_*.

#### 3.3.2. Fitness Measure

The fitness function, Equations (14)–(17) in this work, was utilized to appraise the performance of the new particles generated with each iteration.

#### 3.3.3. Archive Management

The non-dominant solution generated in the iterations was accessed from the archive. If the solution in the archive could be dominated by the new solution, the non-dominant one in the archive was marked and replaced with the new non-dominant solution. If duplicate non-dominant solutions were found, the redundant solutions would be deleted. If the number of non-dominant solutions in the archive exceeded the capacity, the solutions in the archive would be sorted in descending order, and then the remaining 30% of the solutions would be removed.

#### 3.3.4. Leader Selection

a. Selection of *gbest*:

Referring to Ref. [34], the density of solutions in the archive was estimated through the crowding distance sorting method. Firstly, the objective values in Formulas (14)–(17) were sorted separately, and the boundary values of *f_max_* and *f_min_* were ascertained. The crowding distance was retrieved from Formula (20), and then the top 10% of the solutions were selected by descending order. Finally, the particle with the minimum value was found as *gbest*.

(20) $$Y_{i}=\frac{f_{a-1}-f_{a+1}}{f_{max}-f_{\min}}$$

where *f*_*a*−1_ and *f*_*a*+1_ represent the objective values of two adjacent particles.

b. Selection of *pbest*:

If the particle’s current fitness value was lower than the previous one of *pbest*, *pbest* would be updated.

#### 3.3.5. Improved MOPSO

a. Normal distribution decay inertial weight:

The MOPSO had poor global search ability in the early and later stages and might have fallen into the local optimal solution. To solve this problem, the search capability of MOPSO could be improved with the normal distribution decay inertial weight *ω* (Ref. [35]) and determined by the number of iterations. The larger weight *ω* could improve global search ability, while the smaller *ω* might improve local search ability. The *ω* is shown in Formula (21).

(21) $$\omega =\omega_{\min}+(\omega_{\max}-\omega_{\min})\frac{1}{\sqrt{2\pi }\theta }e^{-\frac{{\left(r/r_{\max}\right)}^{2}}{2\theta^{2}}}$$

where *θ* = 0.4433 represents the degree of data dispersion, *ω_max_* = 0.9, *ω_min_* = 0.4, *r* is the current iteration number, and *r_max_* is the maximum iteration number.

b. Sigmoid-based acceleration coefficients:

Referring to Ref. [36], the larger acceleration coefficients, *c*_1_ and *c*_2_, could fly particles to *pbest* and *gbest* faster. The acceleration coefficients *c*_1_ and *c*_2_ changing with time can enhance the initial global search, and particles could converge on the global optimal solution at the end of the search.

When the acceleration coefficients *c*_1_ and *c*_2_ changed in the intervals of [2.5, 0.5] and [0.5, 2.5], respectively, the performance of the algorithm could be significantly improved (Refs. [37,38]). Inspired by this, an acceleration coefficient was deployed to improve performance based on the proposed sigmoid by Ref. [39], whose *c*_1_ and *c*_2_ changed from 2.5 and 0.5 at the initial stage of the search, respectively.

(22) $$c_{1}(r)=\frac{1}{1+\exp(-0.0001*r/r_{\max})}+(c_{end}-c_{ori})*{\left(\frac{r}{r_{\max}}-1\right)}^{2}$$

(23) $$c_{2}(r)=\frac{1}{1+\exp(-0.0001*r/r_{\max})}+(c_{end}-c_{ori})*{\left(\frac{r}{r_{\max}}\right)}^{2}$$

where *c_end_* = 2.5, *c_ori_* = 0.5, and *r* and *r_max_* are the number of iterations and the maximum number of iterations, respectively. 

**Figure 7:**
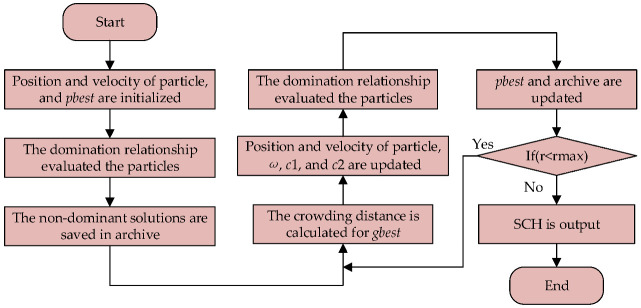
Flow chart of the MOPSO.

The flow chart of the MOPSO is shown in Figure 7. In this procedure, at the start, *pbest*, the particle’s position and speed, were initialized. Then, Formulas (14)–(17) were utilized for the fitness of particles, which were later assessed by the domination relationship, and the qualified solution was sorted and stored in the archive as non-dominated solutions. Following that, crowding distance was predicted for *gbest* to update the position and velocity of the particle; meanwhile, *ω*, *c*_1_, *c*_2_, and the dominant relationship were destined to renew the archive and *pbest*. Finally, the SCHs were output once the iteration criterion was met.

### 3.4. The optimal trajectory of MS

The trajectory length of MS has quite a significant influence on acquisition delay, according to Ref. [40].

The optimal trajectory of MS and the reduction in the data acquisition delay could be regarded as a TSP problem and solved with ACO. All SCHs were regarded as the cities in the TSP problem. *M*2 ants were randomly distributed in *k* CHs and the probability of Ant *q* from node *C_b_* to *C_g_* is estimated with Equation (24):

(24) $$P_{bg}^{q}(h)=\left\{\begin{array}{cc} \frac{{\left[\tau_{bg}(h)\right]}^{\alpha }{\left[\eta_{bg}(h)\right]}^{\beta }}{{\sum_{l\in allowed_{q}}\left[\tau_{bl}(h)\right]}^{\alpha }{\left[\eta_{bl}(h)\right]}^{\beta }} & ,l\in allowed_{q}\\ 0 & ,l\notin allowed_{q} \end{array}\right.$$

where *η_bg_*(*h*) shown in Formula (25) is the heuristic information of *C_b_* and *C_g_*, and *τ_bg_*(*h*) represents the pheromone concentration on the path from *C_b_* to *C_g_* at time h. *α* and *β* are weight factors, *α* is shown in Equation (26), and *allowed_q_* is the set of nodes that Ant *q* has not searched yet.

The traditional ACO has a slow convergence speed and is easy to fall into the local optimal solution. To overcome the defect, the distance heuristic factor in Expression (25) was utilized in this work to enhance the influence on the next node.

(25) $$\eta_{bg}=\frac{1}{\min[dis(b,g)+dis(g,Init)]}$$

where *dis*(*b*, *g*) represents the distance from *C_b_* to *C_g_*, and *dis*(*g*, *Init*) represents the distance from *C_g_* to the initial access node.

A factor reflects the importance of pheromone in the search process, and only affects the convergence speed of the algorithm, so, referring to Ref. [41], the *α* factor is written as Formula (26).

(26) $$\alpha (fre)=\alpha_{0}*(1+e^{-\sigma fre}),\;\;\;0\leq fre\leq fre_{\max}$$

where *α*_0_ represents the basic factor, *σ* represents a random value between 0 and 1, *fre* represents the current number of ACO iterations, and *fre_max_* represents the maximum number of ACO iterations.

Pheromone volatilizing over time is shown in Formula (27).

(27) $$\tau_{bg}(h+H)=(1-\phi )\tau_{bg}(h)+\Delta \tau_{bg}(h,h+H)$$

where volatile factor *Φ* is between 0 and 1, ∆*τ_bg_*(*h*, *h + H*) represents the pheromone increment of the path from node *C_b_* to *C_g_* at time *h* to *h + H*. ∆*τ_bg_*(*h*, *h + H*) is shown in Formula (28):

(28) $$\Delta \tau_{bg}(h,h+H)=\left\{\begin{array}{c} \sum_{q}^{m2}\frac{Q}{L_{q}}\;\mathrm{In}\;\mathrm{the}\;\mathrm{time}\;\mathrm{from}\;h\;\mathrm{to}\;h+H,\;q\;\mathrm{passes}\;\mathrm{through}\;C_{b}\;\mathrm{and}\;C_{g}\\ 0\;\;\;\mathrm{otherwise}\; \end{array}\right.$$

where *m*2 is the number of ants, *Q* is the constant of pheromone intensity, and *L_q_* is the length of the path from *C_b_* to *C_g_* for Ant *q*.

## 4. Simulation and Analysis

We built the simulation environment in MATLAB version 2018b, and we conducted the experiments using a computer with Intel(R) Core(TM) and i7-7500U CPU, with a processor base frequency of 2.70 GHz, 20.00 GB memory, and 4 MB cache.

The PSO-ECHS and PSO-C algorithms refer to Refs. [6,42], the classical LEACH algorithm, its improved algorithm LEACH-C, and Power-Efficient Gathering in Sensor Information Systems (PEGASIS) were compared with the proposed approach.

**Figure 8:**
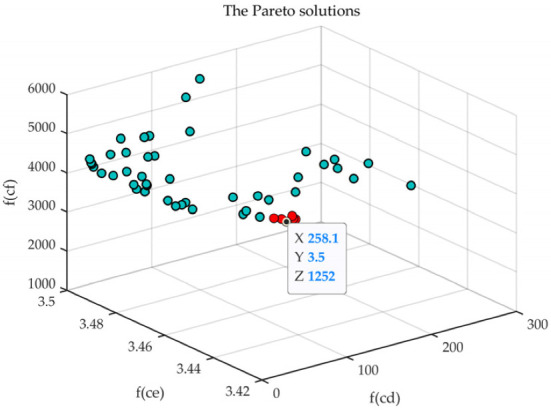
The Pareto solutions.

The boundary conditions of the network are shown in Table 2, and the parameters of MOPSO are shown in Table 3. The Pareto solutions generated via MOPSO are shown in Figure 8, where the red dots are preliminary candidates, the dots in other colors are pareto solutions. Then, the relative distance between CHs screened those candidates for the final black-marked SCH, whose *f_cd_*, *f_ce_*, and *f_cf_* were 258.0601, 3.4996, and 1252.316, respectively.

**Figure 9:**
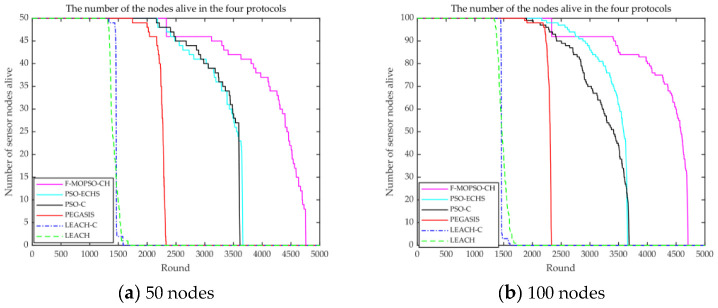
Comparison of the network lifetime for (**a**) 50 and (**b**) 100 nodes.

The comparison of lifetimes among models is shown in Figure 9.

From Figure 9a, for 50 nodes, the dead nodes of the F-MOPSO-CH appeared at about 2330 rounds, while the dead nodes of PSO-ECHS, PSO-C, PEGASIS, LEACH-C, and LEACH blinked at about 2180, 2160, 1740, 1330, and 1310 rounds, and the improvement rates were about 6.9%, 7.9%, 33.9%, 75%, and 77.8%, respectively. From Figure 9b, for 100 nodes, the F-MOPSO-CH showed dead nodes at about 2330 rounds, while those of PSO-ECHS, PSO-C, PEGASIS, LEACH-C, and LEACH occurred at about 2160, 1900, 1850, 1450, and 1330 rounds, and the improvement rates were about 7.9%, 22.6%, 25.9%, 60.6%, and 75%, respectively. It is obvious that the FCM algorithm was used to obtain FCHs, and the distribution of FCHs was very uniform, which is conducive to reducing node energy consumption.

**Figure 10:**
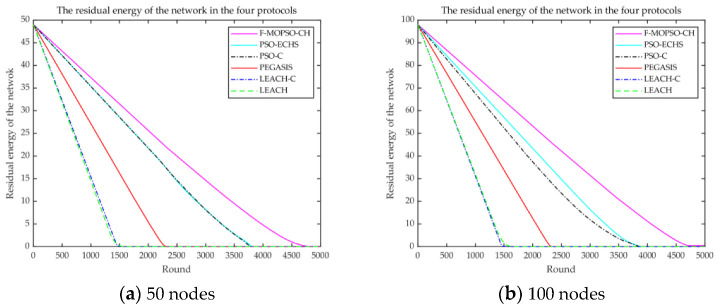
Comparison of the network energy consumption for (**a**) 50 and (**b**) 100 nodes.

The comparison of energy consumption is shown in Figure 10. In Figure 10a, for 50 nodes, the F-MOPSO-CH ran out of energy at about 4750 rounds, while PSO-ECHS, PSO-C, PEGASIS, LEACH-C, and LEACH were out of energy at about 3660, 3680, 2330, 1580, and 1630 rounds, and the network lifetime increased by about 29.8%, 29%, 103.9%, 200.6%, and 191.4%, respectively. In Figure 10b, for 100 nodes, the F-MOPSO-CH drained energy at about 4780 rounds, while PSO-ECHS, PSO-C, PEGASIS, LEACH-C, and LEACH did so at about 3665, 3610, 2330, 1610, and 1670 rounds, and the network lifetime increased by about 30.4%, 32.4%, 105.1%, 196.9%, and 186.2%, respectively. It is obvious that the SCHs obtained with MOPSO can reduce the energy consumption of FCHs so that the energy of the network can be maintained for a longer time.

**Figure 11:**
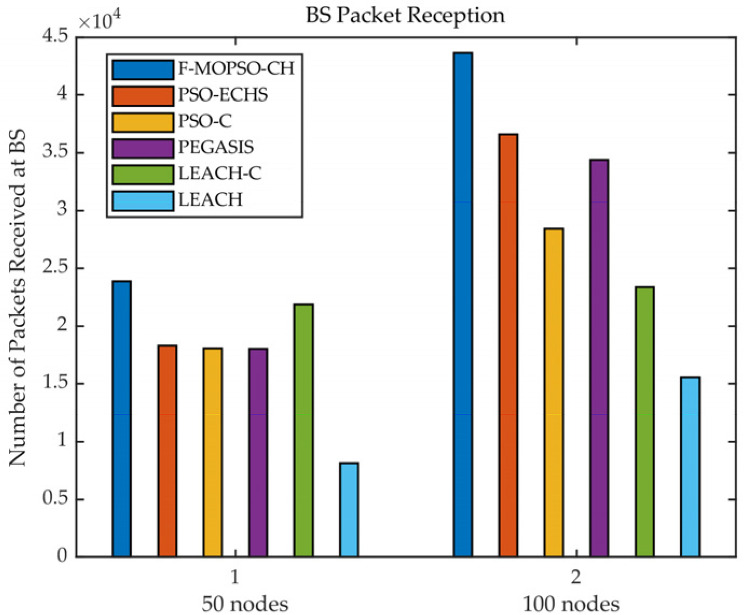
Comparison of BS packet reception.

The comparison of BS packet reception is shown in Figure 11. In Figure 11, for 50 nodes, the number of packets received by F-MOPSO-CH at BS was 23,865, while PSO-ECHS, PSO-C, PEGASIS, LEACH-C, and LEACH were 18,325, 18,070, 18,020, 21,817, and 8118, respectively. The BS packet reception increased by about 30.2%, 32%, 32.4%, 9.3%, and 193.9%, respectively. For 100 nodes, the number of packets received by F-MOPSO-CH at BS was 43641, while PSO-ECHS, PSO-C, PEGASIS, LEACH-C, and LEACH were 36,590, 28,420, 34,358, 23,358, and 15,515, respectively. The BS packet reception increased by about 19.3%, 53.5%, 27%, 86.8%, and 181.2%, respectively. Because F-MOPSO-CH has two kinds of CH working together, it can receive more packets.

**Figure 12:**
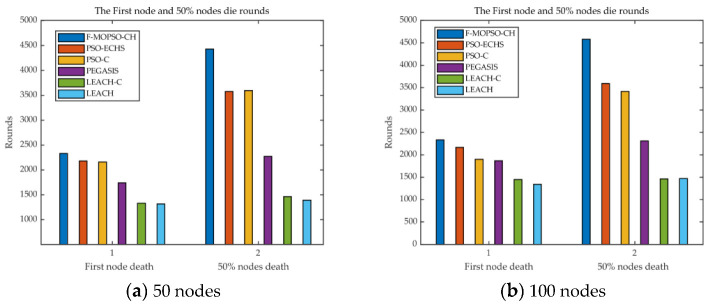
Comparison of the FND and the HND for (**a**) 50 and (**b**) 100 nodes.

The F-MOPSO-CH was compared with PSO-ECHS, PSO-C, PEGASIS, LEACH-C, and LEACH. The first node death (FND) and the 50% node death (HND) are presented in Figure 12.

From Figure 12a, for 50 nodes, compared with PSO-ECHS, PSO-C, PEGASIS, LEACH-C, and LEACH, the approach increased the rounds of FND by 7%, 8.1%, 33.9%, 74.9%, and 77.3%, respectively, and the rounds of HND by 24.7%, 23.3%, 94.7%, 202%, and 217.8%, respectively.

In Figure 12b, for 100 nodes, compared with PSO-ECHS, PSO-C, PEGASIS, LEACH-C, and LEACH, the approach boosted the rounds of FND by 7.9%, 22.9%, 25.1%, 61%, and 74.4%, respectively, and the rounds of HND by 27.8%, 34.2%, 98.3%, 213.1%, and 211.2%, respectively.

**Figure 13:**
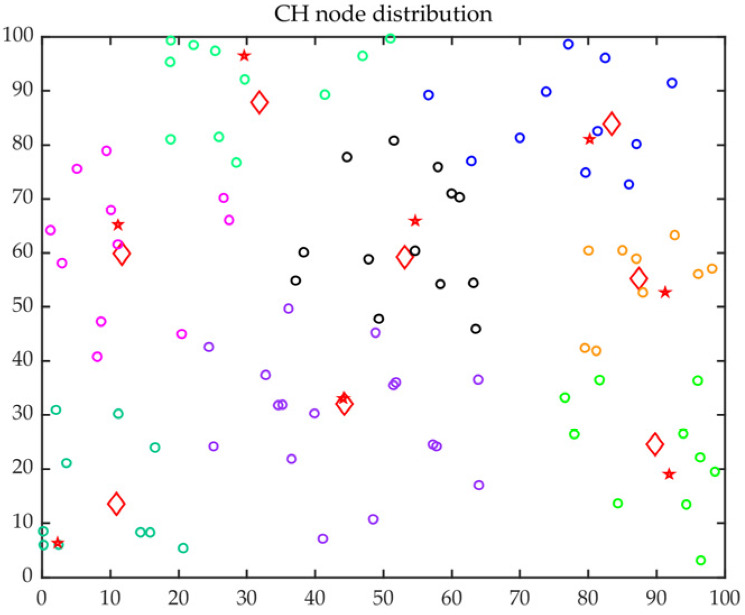
CH distribution.

**Figure 14:**
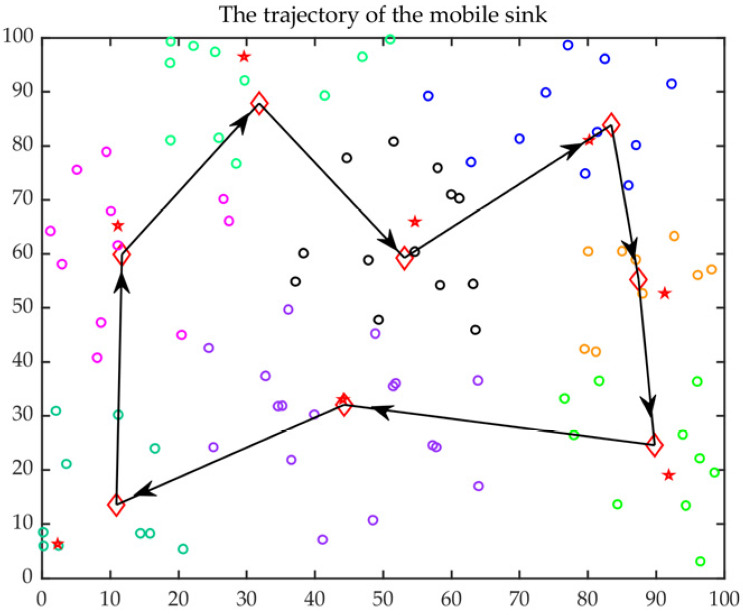
The optimal trajectory of MS.

The improved ACO was applied for the optimal trajectory of MS with a lower delay. The CH distribution and the optimal trajectory of MS are presented in Figure 13 and Figure 14, respectively. In those figures, the red five-pointed stars represent FCHs, the red quadrilateral represents SCHs, the rings with different colors indicate different clustering results, and the black arrow indicates the optimal trajectory. It can be observed that most of the FCHs were distributed in the cluster center, and the distributions of FCHs and SCHs were quite uniform.

**Figure 15:**
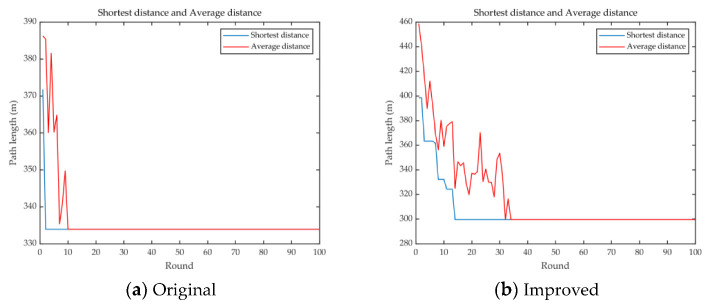
Comparison between original and improved.

The comparison of the trajectory is shown in Figure 15. In Figure 15a, the shortest trajectory before optimization is 332 m, while Figure 15b demonstrates the shortest trajectory of 299 m after optimization. Comparing both, the optimal trajectory was reduced by about 10% with a definite reduction in data acquisition delay.

## 5. Conclusions

In the F-MOPSO-CH algorithm proposed in this paper, the combination of FCM and the optimal number of CH was initially utilized for the confirmation of FCHs. Then, based on those acknowledged results, a MOPSO was applied for the SCHs to overcome the hotspot issue of FCHs. Following that, the improved ACO was used to reduce the data acquisition delay of MS with the reasonable deployment of SCHs. At last, the lifetimes, the death rounds of the first node and the 50% nodes, and the number of packets received at the base station for the F-MOPSO-CH, PSO-ECHS, PSO-C, PEGASIS, LEACH-C, and LEACH were compared, and the improved trajectory of MS was also compared with the original one to validate the effectiveness of the F-MOPSO-CH. 

The main discoveries are concluded as follows:

(a) The combination of the FCM algorithm and the optimal number of CH could produce a more uniform distribution of the FCHs with a reduction in energy consumption.
(b) The application of the MOPSO for SCHs can effectively reduce the load on FCHs.
(c) In the comparison with PSO-ECHS, PSO-C, PEGASIS, LEACH-C, and LEACH algorithms, for 100 nodes, the F-MOPSO-CH could respectively prolong the FND lifetime by 7.9%, 22.9%, 25.1%, 61%, and 74.4%. As for the HND, its lifetime increased by 27.8%, 34.2%, 98.3%, 213.1%, and 211.2%, respectively. The base station packet reception increased by about 19.3%, 53.5%, 27%, 86.8%, and 181.2%, respectively.
(d) In the F-MOPSO-CH for 100 nodes, the improved ACO could shrink the trajectory of MS by a decrease of 10%.

## Figures and Tables

**Table 1:** Formula (6) *k* value results.

Time	1	2	3	4	5	6	7	8	9	10
Results	7.1449	7.5578	7.5839	6.9777	7.3939	7.5878	7.8473	8.1677	7.9214	7.6962

**Table 2:** The boundary conditions of network.

Parameters	Values
Network distribution area	100 × 100 m^2^
Number of nodes (N)	50–100
Location coordinate of the base station	(50,110)
Initial energy	0.98 J
Data packet size	4000 bits
Speed of MS	2 m/s
*E_da_*	5 nJ/bit
Energy consumption on circuit (*E_elec_*)	50 nJ/bit
Free-space channel parameter (*ε_fs_*)	10 pJ/bit/m^2^
Multi-path channel parameter (*ε_mp_*)	0.0013 pJ/bit/m^4^

**Table 3:** Parameters of MOPSO.

Parameters	Values
Size of population	400
Maximum iteration	200
Particle position range	[0, 100]
Particle velocity range	[0, 10]
*r*_1_ and *r*_2_	[0, 1]
*c*_1_ and *c*_2_	[0.5, 2.5]
*ω*	[0.4, 0.9]

## Data Availability

All data are included in the work. No additional data present.

## Nomenclature

ACO	Ant colony optimization algorithm
*allowed_q_*	The set of nodes that ant *q* has not searched yet
BAT	BAT algorithm
BS	Base station
CH	Cluster head
*c*_1_	The acceleration coefficient of MOPSO
*c*_2_	The acceleration coefficient of MOPSO
*c_end_*	*c_end_* = 2.5
*ch*	*ch* = 1, 2,3…*k*
*c_ori_*	*c_ori_* = 0.5
*Dis*(*CN_W_*, *CN_S_*)	The distance between a SCH and other SCHs
*Dis*(*FN_S_*, *CN_S_*)	The distance between a FCH and its corresponding SCH
*Dis*(*SN_X_*, *CN_S_*)	The distance from the non-CH node to the SCH
*Dis*(*SN_X_*, *FN*)	The distance from the non-CH in the cluster to the FCH
*d*	Data transmission distance
*d*_0_	Threshold value
*dis*(*b*, *g*)	The distance from *C_b_* to *C_g_*
*dis*(*g*, *Init*)	The distance from *C_g_* to the initial access node
*d_toBS_*	Average distance between all nodes and the BS
*E_TX_*	Energy consumption of a node for sending data
*E_RX_*	Energy consumption of a node receiving data
*E*(*ch*)	Residual energy of CH
*E*(*x*)	The residual energy of non-CH nodes in the cluster
*F*	Edge length of the sensor square area
FCH	First CH
FCM	Fuzzy c-means
*FN*	A certain cluster head
FND	First node death
*f*_*a*−1_ and *f*_*a*+1_	The objective function values of adjacent particles
*fre*	The current number of ACO iterations
*gbest*	Global leader of MOPSO
HND	50% nodes death
*h_sn_*	The number of nodes in a cluster
HND	50% nodes death
IOT	Internet of things
*J_FCM_*	Objective function of FCM
*k*	The number of cluster heads
LEACH	Low energy adaptive clustering hierarchy
LEACH-C	LEACH-C algorithm
*L_q_*	The length of path from *C_b_* to *C_g_* for Ant *q*
MOPSO	Multi-objective particle swarm optimization
MS	Mobile sink
*m*	Fuzzy weighted index
*m*1	The *m*1 bit of data
*m*2	The number of ants
*n*	Number of sensor node
PEGASIS	PEGASIS algorithm
PSO	Particle swarm optimization
*pbest*	Individual leader of MOPSO
*Q*	Pheromone intensity
*q*	The ants of ACO
*r*	The number of iterations of MOPSO
*r*_1_	Random numbers between [0, 1]
*r*_2_	Random numbers between [0, 1]
SCH	Second CH
*SN*	Sensor node
TSP	Traveling salesman problem
*u_ij_*	Membership degree of node
*V*	Velocity of particle
*v*	Speed of mobile sink
$v_{j}^{o}$	Cluster center
WSN	Wireless sensor networks
*X*	Position of particle
*α*	The weight factors of ACO
*α*_0_	The basic factor
*β*	The weight factors of ACO
*ε_fs_*	Parameter in the power amplifier
*ε_mp_*	Parameter in the power amplifier
*η_bg_*(*h*)	The heuristic information of ACO
*θ*	The degree of data dispersion
λ	The weight of Formula (16)
*σ*	A constant between 0 and 1
*τ_bg_*(*h*)	The pheromone concentration of ACO
∆*τ_bg_*(*h*, *h + H*)	The pheromone increment of ACO
*Ψ*	The weight of Formula (11)
*Φ*	The volatile factor [0, 1]
*ω*	The inertial weight of the MOPSO
*χ*	Index of data transmission distance

## References

[1] Choi H.W., Shin D.W., Yang J., Lee S., Figueiredo C., Sinopoli S., Ullrich K., Jovančić P., Marrani A., Momentè R. (2022). Smart textile lighting/display system with multifunctional fibre devices for large scale smart home and IoT applications. Nat. Commun..

[2] Jha A., Verburg A., Tukkaraja P. (2022). Internet of Things-Based Command Center to Improve Emergency Response in Underground Mines. Saf. Health Work.

[3] Kumar P.M., Hong C.S., Afghah F., Manogaran G., Yu K., Hua Q., Gao J. (2022). Clouds Proportionate Medical Data Stream Analytics for Internet of Things-Based Healthcare Systems. IEEE J. Biomed. Health Inform..

[4] Ramachandran V., Ramalakshmi R., Kavin B.P., Hussain I., Almaliki A.H., Almaliki A.A., Elnaggar A.Y., Hussein E.E. (2022). Exploiting IoT and Its Enabled Technologies for Irrigation Needs in Agriculture. Water.

[5] Dietrich I., Dressler F. (2009). On the lifetime of wireless sensor networks. ACM Trans. Sens. Netw..

[6] Rao P.C.S., Jana P.K., Banka H. (2017). A particle swarm optimization based energy efficient cluster head selection algorithm for wireless sensor networks. Wirel. Netw..

[7] He W. (2019). Energy-Saving Algorithm and Simulation of Wireless Sensor Networks Based of Clustering Routing Protocol. IEEE Access.

[8] Hou J., Qiao J.H., Han X.L. (2022). Energy-Saving Clustering Routing Protocol for Wireless Sensor Networks Using Fuzzy Inference. IEEE Sens. J..

[9] Loganathan S., Arumugam J. (2021). Energy Efficient Clustering Algorithm Based on Particle Swarm Optimization Technique for Wireless Sensor Networks. Wirel. Pers. Commun..

[10] Othman S.B., Bahattab A.A., Trad A., Youssef H. (2014). Confidentiality and Integrity for Data Aggregation in WSN Using Homomorphic Encryption. Wirel. Pers. Commun..

[11] Tam N.T., Dat V.T., Lan P.N., Thanh Binh H.T., Vinh L.T., Swami A. (2021). Multifactorial evolutionary optimization to maximize lifetime of wireless sensor network. Inf. Sci..

[12] Maivizhi R., Yogesh P. (2021). Q-learning based routing for in-network aggregation in wireless sensor networks. Wirel. Netw..

[13] Chang C.Y., Chen S.Y., Chang I.H., Yu G.J., Roy D.S. (2020). Multirate Data Collection Using Mobile Sink in Wireless Sensor Networks. IEEE Sens. J..

[14] Raj P.V.P., Khedr A.M., Aghbari Z.A. (2020). Data gathering via mobile sink in WSNs using game theory and enhanced ant colony optimization. Wirel. Netw..

[15] Wu X., Chen Z., Zhong Y., Zhu H., Zhang P. (2022). End-to-end data collection strategy using mobile sink in wireless sensor networks. Int. J. Distrib. Sens. Netw..

[16] Ali H., Tariq U.U., Hussain M., Lu L., Panneerselvam J., Zhai X.J. (2021). ARSH-FATI: A Novel Metaheuristic for Cluster Head Selection in Wireless Sensor Networks. IEEE Syst. J..

[17] Amutha J., Sharma S., Sharma S.K. (2021). Strategies based on various aspects of clustering in wireless sensor networks using classical, optimization and machine learning techniques: Review, taxonomy, research findings, challenges and future directions. Comput. Sci. Rev..

[18] Rawat P., Chauhan S. (2021). Clustering protocols in wireless sensor network: A survey, classification, issues, and future directions. Comput. Sci. Rev..

[19] Tyagi S., Kumar N. (2013). A systematic review on clustering and routing techniques based upon LEACH protocol for wireless sensor networks. J. Netw. Comput. Appl..

[20] Kumar P., Amgoth T., Annavarapu C.S.R. (2018). ACO-based mobile sink path determination for wireless sensor networks under non-uniform data constraints. Appl. Soft Comput..

[21] Donta P.K., Amgoth T., Annavarapu C.S.R. (2020). An extended ACO-based mobile sink path determination in wireless sensor networks. J. Ambient. Intell. Humaniz. Comput..

[22] Lee S., Kang M., Kim Y., Yoon I., Noh D.K. (2022). Dual-line data collection scheme for efficient mobile sink operation in solar-powered wireless sensor networks. Sustain. Comput. Inform. Syst..

[23] Roy S., Mazumdar N., Pamula R. (2021). An optimal mobile sink sojourn location discovery approach for the energy-constrained and delay-sensitive wireless sensor network. J. Ambient. Intell. Humaniz. Comput..

[24] Sugihara R., Gupta R.K. (2010). Optimal Speed Control of Mobile Node for Data Collection in Sensor Networks. IEEE Trans. Mob. Comput..

[25] Wang J., Cao J., Sherratt R.S., Park J.H. (2017). An improved ant colony optimization-based approach with mobile sink for wireless sensor networks. J. Supercomput..

[26] Tabibi S., Ghaffari A. (2018). Energy-Efficient Routing Mechanism for Mobile Sink in Wireless Sensor Networks Using Particle Swarm Optimization Algorithm. Wirel. Pers. Commun..

[27] Chauhan V., Soni S. (2019). Mobile sink-based energy efficient cluster head selection strategy for wireless sensor networks. J. Ambient. Intell. Humaniz. Comput..

[28] Kaddi M., Banana A., Omari M. (2021). ECO-BAT: A New Routing Protocol for Energy Consumption Optimization Based on BAT Algorithm in WSN. Comput. Mater. Contin..

[29] Wang W., Shi H., Wu D., Huang P., Gao B., Wu F., Xu D., Chen X. (2016). VD-PSO: An efficient mobile sink routing algorithm in wireless sensor networks. Peer-to-Peer Netw. Appl..

[30] Chao F., He Z.Q., Pang A.P., Zhou H.B., Ge J.J. (2019). Path Optimization of Mobile Sink Node in Wireless Sensor Network Water Monitoring System. Complexity.

[31] Anand V., Pandey S. (2020). New approach of GA-PSO-based clustering and routing in wireless sensor networks. Int. J. Commun. Syst..

[32] Benmahdi M.B., Lehsaini M. (2020). Performance evaluation of main approaches for determining optimal number of clusters in wireless sensor networks. Int. J. Ad Hoc Ubiquitous Comput..

[33] Zhang B., Jiang H. (2020). Research on clustering method of wireless sensor network based on bisecting K-means. J. Hefei Univ. Technol. (Nat. Sci.).

[34] Deb K., Pratap A., Agarwal S., Meyarivan T. (2002). A fast and elitist multiobjective genetic algorithm: NSGA-II. IEEE Trans. Evol. Comput..

[35] Xu H., Ji W., Sun X., Luo Q. (2020). A PSO algorithm with inertia weight decay by normal distribution. J. Shenzhen Univ. (Sci. Eng.).

[36] Ratnaweera A., Halgamuge S.K., Watson H.C. (2004). Self-organizing hierarchical particle swarm optimizer with time-varying acceleration coefficients. IEEE Trans. Evol. Comput..

[37] Chen K., Zhou F., Wang Y., Yin L. (2018). An ameliorated particle swarm optimizer for solving numerical optimization problems. Appl. Soft Comput..

[38] Chen K., Zhou F., Yin L., Wang S., Wang Y., Wan F. (2018). A hybrid particle swarm optimizer with sine cosine acceleration coefficients. Inf. Sci..

[39] Tian D., Zhao X., Shi Z. (2019). Chaotic particle swarm optimization with sigmoid-based acceleration coefficients for numerical function optimization. Swarm Evol. Comput..

[40] Mehto A., Tapaswi S., Pattanaik K.K. (2021). Multi-objective particle swarm optimization based rendezvous point selection for the energy and delay efficient networked wireless sensor data acquisition. J. Netw. Comput. Appl..

[41] Ling C., Sun W. (2019). Wireless sensor network routing based on improved ant colony algorithm. Comput. Eng. Des..

[42] Latiff N., Tsimenidis C.C., Sharif B.S. Energy-Aware Clustering for Wireless Sensor Networks using Particle Swarm Optimization. Proceedings of the 2007 IEEE 18th International Symposium on Personal, Indoor and Mobile Radio Communications.

